# Sublingual immunotherapy in allergic asthma: Current evidence and needs to meet

**DOI:** 10.4103/1817-1737.65038

**Published:** 2010

**Authors:** Cristoforo Incorvaia, Gian Galeazzo Riario-Sforza, Stefano Incorvaia, Franco Frati

**Affiliations:** *Allergy/Pulmonary Rehabilitation, ICP Hospital, Milan, Italy*; 1 *Institute of Pediatrics, University, Department of Medical and Surgical Specialties and Public Health, Perugia, Italy*

**Keywords:** Allergic asthma, efficacy, specific immunotherapy, sublingual immunotherapy, safety

## Abstract

Allergen-specific immunotherapy is aimed at modifying the natural history of allergy by inducing tolerance to the causative allergen. In its traditional, subcutaneous form, immunotherapy has complete evidence of efficacy in allergic asthma. However, subcutaneous immunotherapy (SCIT) has a major flaw in side effects, and especially in possible anaphylactic reactions, and this prompted the search for safer ways of administration of allergen extracts. Sublingual immunotherapy (SLIT) has met such need while maintaining a clinical efficacy comparable to SCIT. In fact, the safety profile, as outlined by a systematic revision of the available literature, was substantially free from serious systemic reactions. A number of meta-analyses clearly showed that SLIT is effective in allergic rhinitis by significantly reducing the clinical symptoms and the use of anti-allergic drugs, while the efficacy in allergic asthma is still debated, with some meta-analyses showing clear effectiveness but other giving contrasting results. Besides the efficacy on symptoms, the preventive activity and the cost-effectiveness are important outcomes of SLIT in asthma. The needs to meet include more data on efficacy in house dust mite asthma, optimal techniques of administration and, as previously done with SCIT, introduction of adjuvants able to enhance the immunologic response and use of recombinant allergens.

Allergen-specific immunotherapy, in its traditional, subcutaneous form, has complete evidence of efficacy in allergic asthma, as demonstrated by a meta-analysis of 67 double-blind, placebo-controlled studies showing a significant effectiveness on asthmatic symptoms and bronchial hyperresponsiveness.[1] However, subcutaneous immunotherapy (SCIT) has a major flaw in side effects, and especially in systemic reactions of the anaphylactic kind, that are quite rare but may be life-threatening and even fatal.[2] This prompted the search for safer ways of administration of allergen extracts, and sublingual immunotherapy (SLIT), which was introduced in the 1990s, finally met such need while maintaining a clinical efficacy comparable to SCIT.[3]

In particular, the safety profile, as outlined by a systematic revision of the available literature, was substantially free from serious systemic reactions,[4] though recent data showed that starting SLIT directly with the maintenance dose in patients with previous reactions to SCIT may cause severe reactions also to sublingual administration.[5]

A number of meta-analyses clearly showed that SLIT is effective in allergic rhinitis by significantly reducing the clinical symptoms and the use of anti-allergic drugs,[6–10] but the efficacy in allergic asthma is still debated. In fact, in the first meta-analysis, there were insufficient data from patients with asthma,[6] and subsequent analysis gave contrasting results, some even suggesting negative conclusions.[8,11]

Still, meta-analyses explore the central issue of a medical treatment, that is the efficacy on clinical symptoms, but also other issues are important to define its value. Concerning allergen immunotherapy, a key topic is the ability to modify the natural history of allergy by preventing the development of new sensitizations or the worsening of the disease and by acting even after discontinuation of the treatment; the latter factor being related to the mechanisms of action of immunotherapy. Cost-effectiveness is another important topic to consider, which was recently analyzed in properly designed studies.

## Methods Used for Locating, Selecting, Extracting and Synthesizing Data

Articles on the clinical and immunologic effects of SLIT on allergic asthma were located in PubMed and EMBASE by using the keywords ‘sublingual immunotherapy’, ‘allergic asthma’, ‘meta-analysis’, ‘efficacy’, ‘mechanism of action’ and ‘cost-effectiveness’. Seven meta-analyses evaluating SLIT efficacy in asthma were retrieved. Additional articles were selected because of their addressing particular issues of SLIT in patients with allergic asthma.

## Effects of SLIT on Asthmatic Symptoms and Drug Consumption

These are the outcomes investigated in the meta-analyses on controlled trials. The first meta-analysis on SLIT in asthma was conducted by Olaguibel *et al*. and included seven randomized, controlled studies on children aged up to 14 years.[7] By using the Cochrane method based on calculation of the standardized mean difference (SMD) between actively and placebo-treated patients, the authors found that SLIT was significantly effective on asthma symptoms (SMD – 1.42, *P* = 0.01) and on drug consumption (SMD – 1.01, *P* = 0.06).

In 2006, a meta-analysis on the efficacy of SLIT in asthma included 25 studies with an overall number of 1706 patients.[8] Calculating the SMD, the reduction of asthmatic symptoms did not reach the statistical significance, but using the intention-to-treat method for outcome measures, significant decreases of asthma symptoms and drug consumption and significant improvements of lung function and bronchial hyperreactivity were detected. Also the number needed to treat (NNT) – i.e. the number of patients to be treated to have one patient with significant improvement – was calculated, and the results was 3.7, that is in the range of those reported for injective SIT in asthmatic and rhinitic patients.

Another meta-analysis considering 9 studies on pediatric patients, with a total number of patients corresponding to 441, 232 actively treated and 209 placebo-treated, reported a significant reduction in both symptoms scores (SMD – 1.14, *P* = 0.02) and drug consumption (SMD – 1.63, *P* = 0.007).[10]

A recognized limit of meta-analysis is the relevant heterogeneity of the included studies, mainly due to different scoring systems. Recent evaluations considered altogether the meta-analyses but reached contrasting conclusions. According to Nieto *et al*, the meta-analyses, by checking the data reported in the original studies, show ‘discrepancies, inconsistencies and lack of robustness’ and ‘do not provide enough evidence’ for current routine use of SLIT in patients with allergic asthma.[11] By contrast, the overall evaluation of all meta-analyses (5 on SLIT and 2 on SCIT) by Compalati *et al*,, despite a significant heterogeneity of studies and one negative meta-analysis, lead the authors to conclude that ‘SIT can be recommended for the treatment of respiratory allergy because of its efficacy in reducing asthma and rhinitis symptoms’.[12] However, the major effects on asthma were achieved with the subcutaneous route.

These data clearly suggest that some criticism on the therapeutic role of SLIT in allergic asthma may be reasonable. An objective and updated review by Larenas-Linnemann concluded that there is evidence for a clear effect in pollen-induced asthma, while there is yet room for investigations on SLIT in asthma, especially concerning optimal dosing for dust mites.[13] A further meta-analysis examined 9 studies dealing with mite-induced asthma and found a reduction of symptoms (SMD – 0.95, *P* = 0.02) in 243 patients (adults and children) receiving SLIT compared to 209 receiving placebo. A reduction in rescue medication use was also found (SMD – 1.48, *P* = 0.02).[14] A relevant inter-study heterogeneity was detected, that warrants for large population-based high-quality studies and validated and agreed objective outcomes.

Another possible fruitful approach could be to assess the effects of SLIT on asthma using the tools of the Global Initiative on Asthma (GINA) international guidelines.[15] By this approach it was recently demonstrated that SLIT is able to induce a stepdown of seasonal asthma in grass–pollen allergic patients.[16]

## Preventive Capacity of SLIT

As previously demonstrated for SCIT,[17] SLIT showed the ability to prevent the development of new sensitizations, and the onset of asthma in subjects with rhinitis. The latter capacity was first demonstrated in a study on children with allergic rhinoconjunctivitis treated with co-seasonal SLIT with a grass–pollen extract, in whom a reduced development of asthma was observed in respect to control subjects.[18] Confirmation was offered by a study on 216 children with allergic rhinitis, who were randomized to receive drugs alone or drugs plus SLIT for 3 years. The clinical score was assessed yearly during allergen exposure. Pulmonary function testing and methacholine challenge were performed at the beginning and end of the study; 144 children received SLIT and 72 received drugs only. New sensitizations appeared in 34.8% of controls and in 3.1% of SLIT patients (odds ratio, 16.85 ). Mild persistent asthma was less frequent in SLIT patients. The number of children with a positive methacholine challenge result decreased significantly after three years only in the SLIT group.[19]

The preventive effects of SLIT continue even after its stopping: in a survey over a mean follow-up of 11.6 months after the end of treatment, 80.8% of patients still maintained the previously achieved benefits. During the follow-up period, only 1% of non-asthma patients reported an onset of respiratory symptoms, and only 9.6% of patients showed new sensitizations. All the clinical benefits were strongly linked to the length of treatment: patients with long-lasting benefits were treated for a mean length of 29.1 months, while patients showing a return to pre-SLIT condition were treated for a mean 13.3 months.[20] The long-lasting effects of SLIT were further demonstrated in a prospective study on patients with allergic asthma due to mites, who were divided into two matched groups: 35 underwent a 4- to 5-year course of SLIT with standardized extract and 25 received only drug therapy. The patients were evaluated at three time points (baseline, end of SLIT and 4 to 5 years after SLIT discontinuation) regarding presence of asthma and use of anti-asthma drugs. The SLIT group showed a significant difference versus baseline for the presence of asthma (*P* = 0.001) and the use of asthma medications (*P* = 0.01), whereas no difference was observed in the control group.[21] These findings demonstrated that SLIT maintains the clinical efficacy for 4 to 5 years after discontinuation, such outcome being related to the immunological modifications induced by the treatment.

## Mechanisms of Action of SLIT

**Figure 1:**
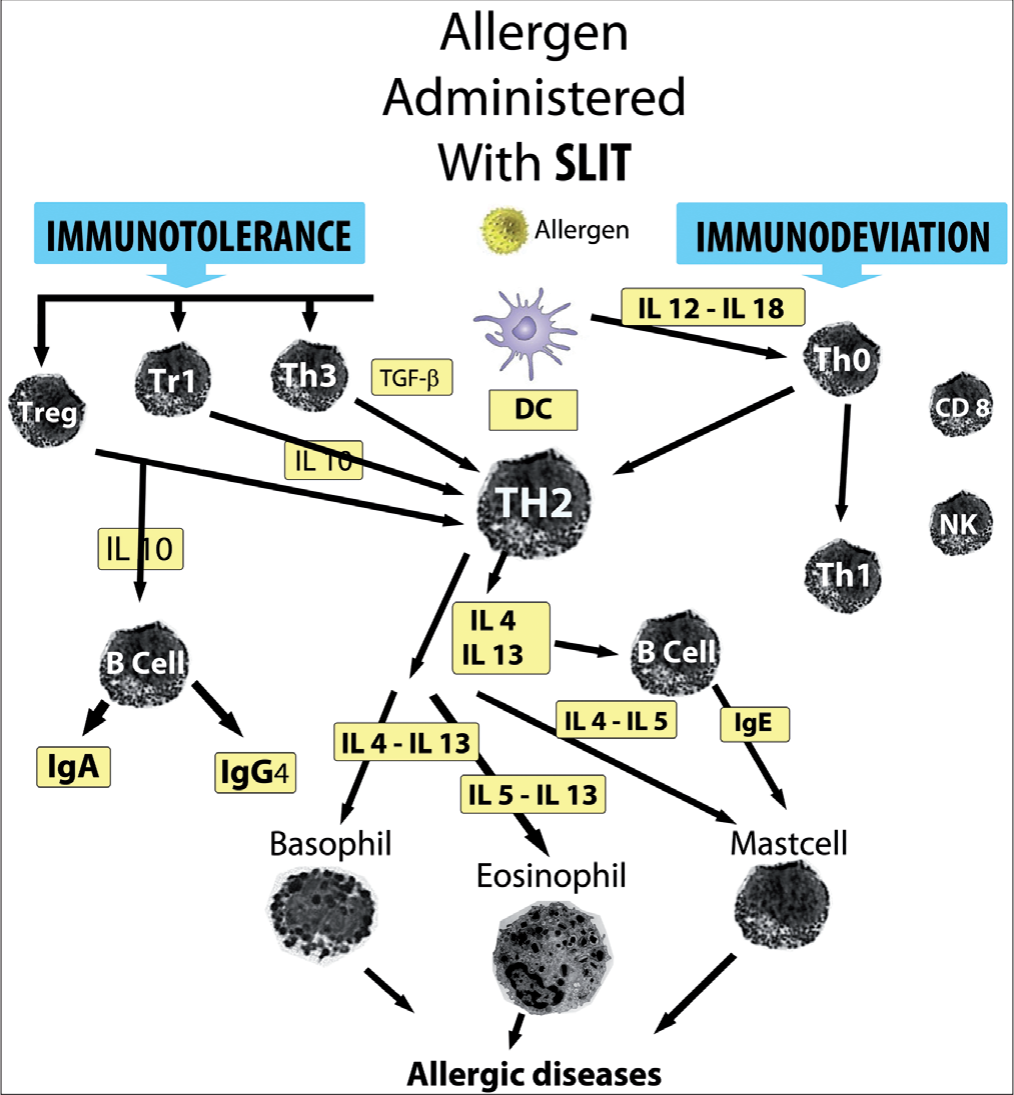
Mechanisms of action of sublingual immunotherapy

Also in this issue, a bulk of data was previously accumulated with SCIT.[22] In the past it was believed that SLIT had different mechanisms of action, but now it has been recognized that the two routes of administration share similar abilities.[23,24] The pivotal action is the antiinflammatory effect of immunotherapy, including SLIT, based on the ability to modify the phenotype of T cells, which in allergic subjects is characterized by a prevalence of the Th2 type, with production of IL-4, IL-5, IL-13, IL-17 and IL-32 cytokines.[25] The immunotherapy-induced changes result in a Th1-type response (immune deviation) related to an increased IFN-gamma and IL-2 production or by a Th2 reduced activity, through a mechanism of anergy or tolerance. It is now known that T-cell tolerance is characterized by the generation of allergen-specific T regulatory (Treg) cells, which produce cytokines such as IL-10 and TGF-beta with immunosuppressant and/or immunoregulatory activity.[26] A prominent role in SLIT is played by dendritic cells in the oral mucosa, which are of critical importance in inducing tolerance to antigens.[27] The tolerance patterns – that are promoted by dendritic cells and driven by Treg - account for the suppressed or reduced activity of inflammatory cells and for the isotypic switch of antibody synthesis from IgE to IgG, and especially to IgG4.[28] The mechanisms promoted by SLIT are summarized in Figure 1. Moreover, data obtained from biopsies clearly indicate that the pathophysiology of the oral mucosa plays a pivotal role in inducing tolerance to the sublingually administered allergen, as showed by subjects treated with high-dose SLIT who have a very low number of mast cells and eosinophils – the effector cells of allergic reactivity – both in the epithelium and sub-epithelium layers, and show insignificant changes after SLIT.[29]

## Cost-effectiveness of SLIT in Allergic Asthma

The immunological effects of SLIT, that underlie the modification of the natural history of respiratory allergy, also account for its cost-effectiveness. In fact, in the initial phases of SLIT the cost of the treatment adds to the cost of symptomatic drugs, but when clinical efficacy takes place the drugs consumption becomes lower and lower. The maximum cost-effectiveness is achieved when SLIT – after 3 to 5 years of treatment – is discontinued but the clinical efficacy is maintained over time. The first published study dealt with the evaluation of cost effectiveness of SLIT in children with allergic rhinitis and asthma, assessed by direct costs (drugs, specialists visits and SLIT) and indirect costs (costs resulting from children school and parental work loss) indicating that high dose SLIT may be effective in reducing the global cost of allergic rhinitis and asthma.[30]

An overall number of 135 patients were analysed. The average annual cost/patient was €2672 before SLIT initiation and €629/year during SLIT. The asthma analysis involved 41 children with SLIT and 35 controls, and showed a substantial reduction in outcomes, though the direct cost per patient over the 4 years follow-up was €1182 for SLIT-treated children and €1100 for controls. These initial findings showed that high-dose SLIT may be effective in reducing the global cost of allergic rhinitis and asthma and comparably expensive to conventional drug treatment in children with allergic asthma over a 4-year follow-up. Another study evaluated the economics of SLIT in patients with pollen allergy and suffering from allergic rhinitis alone or associated with asthma compared with standard case controls.[31] This study was made by a longitudinal observational database operated by a network of allergy centers. Patients were randomly assigned to SLIT (plus drugs as needed) or to treatment with drugs alone. The outcome measures included use of: drugs, SLIT, visits and tests. The results showed that the overall per patient yearly cost of treatment was higher in SLIT patients, both in the whole sample (€311 vs. 180/patient), in rhinitis (€288 vs 116) and rhinitis associated with asthma (€362 vs €230) sub-groups. Patients with rhinitis plus asthma generated more costs than rhinitis alone in both groups. Nevertheless, considerable savings were obtained in the cost of symptomatic drugs (22% for rhinitis, 34% for rhinitis plus asthma) in SLIT patients, thus focusing the use of symptomatic drugs as an important indicator of effective allergy control. However, the most remarkable findings were obtained in a recent study that evaluated the cost-effectiveness of SLIT in patients with mite-induced asthma.[32] A higher mean annual cost was found in the first year in subjects treated with SLIT plus the needed symptomatic drugs compared with subjects only receiving drug treatment, but an economic advantage was evident in the ensuing years and especially when SLIT was discontinued after three years, due to the persistence of good clinical control in SLIT-treated patients.

## Needs to Meet

Sublingual immunotherapy (SLIT) has received extensive demonstration of effectiveness and safety and is currently considered a true option to traditional SCIT to treat respiratory allergy.[33] However, most evidence regarding efficacy, best regimens of administration and optimal dosage was thus far obtained for seasonal allergy due to sensitization to pollens, while data concerning perennial allergens are less than satisfactory and, concerning animal epithelia and moulds (that were demonstrated effective with SCIT),[34] still lacking. As far as house dust mite allergy is concerned, a recent meta-analysis of 9 controlled studies on SLIT in mite-induced asthma showed a significant reduction in symptoms (*P* = 0.02) and in rescue medication (*P* = 0.04), but the overall number of patients from these studies was 243 for active treatment and 209 for placebo (i.e. a relatively limited population), and a relevant inter-study heterogeneity was detected. This leads the authors to state that there is promising evidence of efficacy for SLIT, using mite extract in patients suffering from asthma, but more data are needed, derived from large population-based high-quality studies, and corroborated by objective outcomes.[35]

Other fields of research to develop the performances of SLIT concern: the technique of administration, such as the use of muco-adhesive formulations improving the contact of the allergen extract with the oral mucosa;[36] the introduction of adjuvants able to enhance the immunologic response in a tolerogenic direction, for example agents inducing the production of IL-10 such as Lactobacillus plantarum or the combination of 1,25-dihydroxyvitamin D3 plus dexamethasone;[37] the use of recombinant allergens, which can be produced with well-known methods[38] and have demonstrated efficacy with SCIT,[39] but were not yet tested with SLIT despite an adequate theoretical background being available.[40]
